# Time Mating Guinea Pigs by Monitoring Changes to the Vaginal Membrane throughout the Estrus Cycle and with Ultrasound Confirmation

**DOI:** 10.3390/mps4030058

**Published:** 2021-08-27

**Authors:** Rebecca L. Wilson, Kristin Lampe, Brad J. Matushewski, Timothy R. H. Regnault, Helen N. Jones

**Affiliations:** 1 Center for Research in Perinatal Outcomes, College of Medicine, University of Florida, Gainesville, FL 32610, USA; jonesh@ufl.edu; 2 Department of Physiology and Functional Genomics, College of Medicine, University of Florida, Gainesville, FL 32610, USA; 3 Center for Fetal and Placental Research, Cincinnati Children’s Hospital and Medical Center, Cincinnati, OH 45229, USA; Kristin.Lampe@cchmc.org; 4 Department of Kinesiology, Western University, London, ON N6A 5B9, Canada; bjmatush@uwo.ca; 5 Departments of Physiology and Pharmacology, and Obstetrics and Gynaecology, Schulich School of Medicine and Dentistry, Western University, London, ON N6A 5B9, Canada; tim.regnault@uwo.ca; 6 Children’s Health Research Institute and Lawson Health Research Institute, London, ON N6A 5B9, Canada

**Keywords:** guinea pig, pregnancy, time mate, methodology, reproduction

## Abstract

One of the greatest challenges to the development and implementation of pregnancy therapeutics is the ability to rigorously test treatments in clinically relevant animal models. Guinea pigs offer a unique advantage in studying the placenta, fetal development, and reproductive health as they have similar developmental milestones to humans, both throughout gestation and following birth. Tracking the guinea pig estrus cycle is imperative to ensuring appropriately timed mating and can be performed by monitoring the guinea pig vaginal membrane. Here, we describe a methodology to efficiently and accurately time mate guinea pigs, and provide a picture representation of changes to the guinea pig vaginal membrane throughout the estrus cycle. Utilization of this monitoring enabled a 100% pregnancy success rate on the first mating attempt in a cohort of five guinea pigs. This approach, along with early pregnancy ultrasounds as a secondary method to confirm pregnancy, offers a reliable approach to timed mating in the guinea pig.

## 1. Introduction

The placenta is a transient organ, unique to pregnancy which functions to support the passage of oxygen and nutrients to the developing fetus and maintain adaptation of the maternal environment to pregnancy [1], without it, there is no pregnancy. Inappropriate placental development and function is associated with numerous obstetric complications which affect one in four pregnancies worldwide [2]. Conditions such as preeclampsia, stillbirth, and preterm birth contribute significantly to both maternal and neonatal morbidity and mortality. As such, there is a heightened need to continue to develop robust predictive models, diagnostic techniques, and potential therapies for use during pregnancy. However, studying and utilizing pregnant women is considered high risk and consequently, most pregnancy research utilizes animal models from small rodent species to larger mammals such as sheep.

The usefulness of the guinea pig in pregnancy research is often overlooked and more readily performed in mice due to their shorter gestation, lower costs, and more rapid advancement of transgenic model development. However, guinea pigs have smaller litters and deliver precocial young (they are born fully-furred with well-developed sensory and locomotory abilities) [3]. Furthermore, guinea pigs have some similar developmental milestones to humans both throughout gestation and following birth [3,4]. This includes significant fetal fat deposition in late pregnancy [5] and comparable biochemical and morphological development of fetal organs including lungs [6], kidneys [7], and the cardiovascular system [8]. Unlike rats and mice, the type of placenta in the guinea pig reflects the human as they have a haemomonochorial placental barrier: a single syncytiotrophoblast layer separates the maternal blood space from the fetal vessels [9]. Placental establishment is also more similar to humans, with guinea pigs exhibiting deeper placental trophoblast invasion than mice [10] and changes to the maternal hormonal profile throughout pregnancy more closely resembles changes in humans when compared to other rodent species [11]. Altogether, these outcomes make utilizing the guinea pig in pregnancy research superior, particularly for the advancement of understanding the potential mechanisms underlying poor obstetrical outcomes. This knowledge can then be used to facilitate the development of potential therapies to treat obstetrical complications that significantly contribute to long-term morbidity and mortality for mother and child.

One of the principal challenges associated with using guinea pigs for reproductive discoveries is the ability to time their matings. Guinea pigs have an approximately 16-day estrus cycle [12] and 65–70 day gestation [13]. Unlike other rodent species, the formation of a copulatory plug by male guinea pigs after intercourse is debated [14] and as such, it is difficult to ascertain when mating has occurred in order to track pregnancy progression. Some have described methods in which time mating guinea pigs is achieved by allowing females to carry a pregnancy and then breed in 24 h immediately after birth [15]. Others simply allow a time range of plus/minus 1 week based on the length at which the female is housed with the male [16]. However, these methodologies introduce significant variability (mean fetal weight increases from 2.5 ± 0.31 g at GD33 to 12.0 ± 0.77 g at GD39; R. Wilson unpublished) and time which ultimately increases costs associated with performing experiments with guinea pigs. Similar to other rodents, guinea pigs have a four-stage estrus cycle: Estrus, metestrus, diestrus, and proestrus [12]. Estrogen is the dominant hormone during the estrus period and occurs during ovulation, whilst progesterone dominates the diestrus period [17]. All the stages of the estrus cycle can be monitored through vaginal cytological smears. However, these can be time consuming and require experience in understanding the technique. Vaginal impedance can also be used to monitor estrus cycles progression [18], but is also another technique that requires specialized tools. Monitoring changes in the vaginal membrane, which perforates spontaneously at estrus is an easy method to achieve time mated guinea pigs, first described in [14,19]. Herein, we describe this methodology in a simple to follow protocol with photographic evidence of the changes to the vagina membrane, as well as highlight the use of ultrasound in early pregnancy (gestational day 21–30) to definitively confirm the pregnancy.

## 2. Experimental Design

### 2.1. Materials

- Female and male Hartley guinea pigs, between 2–4 months of age (Charles River, Wilmington, MA, USA).
- Standard Guinea Pig Chow with stabilized Vitamin C. Example: LabDiet 5025 (LabDiet, St. Louis, MI, USA; Cat. no. 5025).
- Ultrasound coupling gel. Example: Aquasonic Clear (Parker Laboratories Inc, Fairfield, NJ, USA; Cat. no. 03-08).

### 2.2. Equipment

- Ultrasound machine. Example: Voluson I Portable Ultrasound Machine (GE Healthcare, Boston, MA, USA).
- Ultrasound Probe capable of transmitting at depths of 3–10 cm. Example: 25 E 12 MHz vascular probe (GE Healthcare, Boston, MA, USA).

## 3. Procedure

### 3.1. Animal Husbandry and Daily Estrus Checks

**Figure 1:**
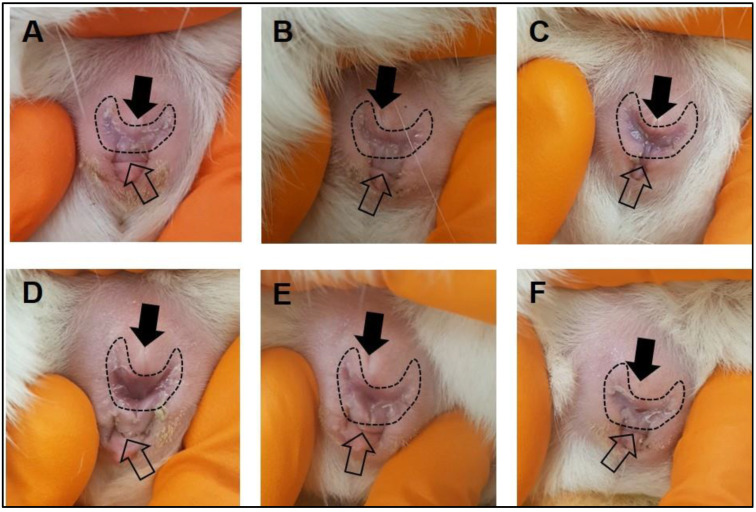
Monitoring of the guinea pig vaginal membrane. During the latent period of the estrus cycle, the guinea pig vaginal membrane (dashed outline) is visibly closed (**A**,**B**). Four to five days prior to ovulation, changes to the color of the vaginal membrane can be observed indicating potential membrane perforation (**C**). At the time of ovulation, the vaginal membrane perforates and increased vaginal secretions/mucus can be observed (**D**). Following ovulation, the vaginal membrane begins to close (**E**,**F**). The closed arrow indicates the urethral opening, the open arrow indicates the anus.

1. Purchase guinea pigs at 500–550 g.
2. Group house guinea pigs of same sex on a 12:12 h light on/off cycle at 22 °C/72 °F and 50% humidity.
3. Provide water and food ad libitum.
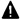
**CRITICAL STEP:** Ensure vitamin C is stabilized within the feed, otherwise, vitamin C will need to be supplemented in the drinking water (400 mg/L).
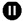
**PAUSE STEP:** Allow guinea pigs to acclimatize in the new location with minimal handling for at least 1 week.
4. After the acclimatization period, make daily observations of the female vaginal membrane by holding the animal secure and using the thumb and index finger to gently manipulate the vaginal opening (Figure 1). Record whether the vaginal membrane appears “closed”, “open”, “opening” or “closing”.
5. Repeat observations daily, in the morning at approximately the same time of day until the vaginal membrane is observed as perforated (Figure 1). Record the date that vaginal membrane is first observed as perforated.

### 3.2. Time Mating Guinea Pigs

1. If housed together, 2–3 days prior to starting the mating, separate male guinea pigs into individual cages.
2. Fourteen days after the vaginal membrane is first observed as perforated, place the female with the male. This will allow the breeding pair to become acquainted before ovulation occurs, leading to a more successful mating. The cage size allowing, up to two females can be placed with a single male.
3. Continue to monitor the vaginal membrane daily, as outlined in Section 3.1 of Step 4.
4. The morning when the vaginal membrane is observed as perforated, record this date as gestational day (GD) 1. Ovulation is presumed to have occurred overnight and is often associated with the presence of ‘estrus fluid’ in the vaginal opening [14]. Record any ‘signs of mating’ observations such as a pale crustiness around the vaginal opening or a vaginal plug.
5. Keep the female(s) with the male until the vaginal membrane closes, females will typically be with males 5–6 days in total.
6. Return the female to the group housing.

### 3.3. Ultrasound Pregnancy Confirmation

1. Ultrasound pregnancy confirmation can be performed without anesthesia [20] between GD21-30.
2. Whilst carefully restraining the guinea pig, shave the female guinea pig’s lower abdomen.
3. Set the ultrasound to a depth between 6 and 10 cm and a frequency between 8 and 10 MHz.
4. Place enough ultrasound coupling gel on the end of the ultrasound probe to cover the probe and perform the ultrasound. Scan from the cervix up to each uterine horn until a conceptus is found. Multiple conceptuses may be observed, but only one is needed to confirm the pregnancy.
5. Once the pregnancy is confirmed, return the animal to the group housing.
6. **OPTIONAL STEP:** Ultrasound pregnancy confirmation can be performed under anesthesia using 4–5% isoflurane mixed with 2 L/min oxygen and maintenance at 1 L/min.
7. If the female is not confirmed pregnant, repeat the process until a successful pregnancy is established.

## 4. Expected Results

**Figure 2:**
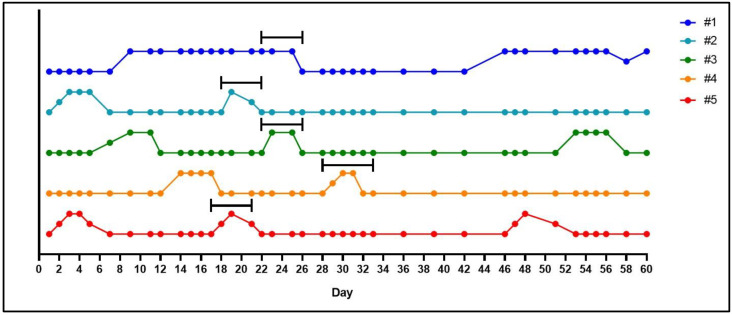
Graphical representation of the estrus cycle in the guinea pig based on monitoring of the vaginal membrane. In five animals, the vaginal membranes were monitored daily and observed as either ‘closed’, ‘open’, ‘opening’ or ‘closing’. A closed vaginal membrane was designated as the baseline and perforation of the membrane was marked as an increase away from the baseline. Females were placed with a male 14 days after perforation of the vaginal membrane was first observed, as indicated by the back bars above the colored lines. Monitoring throughout pregnancy also occurred, in which the vaginal membrane was observed as perforated in two (#3 and #5) out of the five animals. For female #1, observations of a perforated vaginal membrane on day 46 also included blood in the vaginal lumen and a decrease in maternal weight gain. A later ultrasound confirmed the loss of pregnancy in this animal.

Through monitoring changes to the vaginal membrane, we achieved a 100% pregnancy success rate with first time mating of five female guinea pigs. During the latent period of the estrus cycle, the vaginal membrane will appear as a pale color and visibly closed (Figure 1A,B). In the 4–5 days prior to ovulation, the vagina will undergo noticeable changes in color, becoming progressively darker pink, mild swelling of the vulva can also be noted prior to perforation (Figure 1C). Full perforation of the vagina happens within 24–72 h and is noticeable as a complete opening of the vagina, often associated with increased vaginal secretions/mucous (Figure 1D). The vaginal membrane then closes within 48–72 h of full perforation (Figure 1E,F). Based on these observations, we confirmed the guinea pig estrus cycle to be 15–16 days (*n* = 4 out of five female guinea pigs) with observations of initial perforation through to closing of the vaginal membrane lasting between 3–6 days (Figure 2). This was the exception for one female whose vaginal membrane remained perforated for approximately 2 weeks after initially being observed as open. Despite this, she was still placed with a male as described in Section 3.

**Figure 3:**
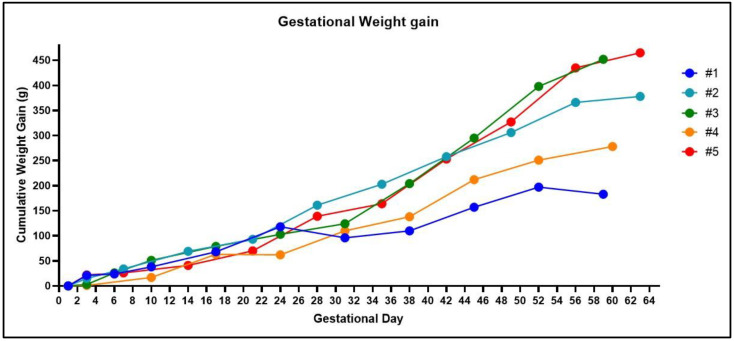
Weight changes in the guinea pig throughout gestation. Noticeable increases in gestational weight are not reliable until mid-pregnancy in the guinea pig. Healthy fetuses were confirmed in all the animals at gestational day 30 using an ultrasound, except for one animal (#1) in which blood in the vaginal lumen was observed at gestational day 24 and was associated with a decrease in cumulative weight gain. At post mortem, #3 had four fetuses, #5 had three fetuses, #2 and #4 had two fetuses, and #1 was not pregnant.

**Figure 4:**
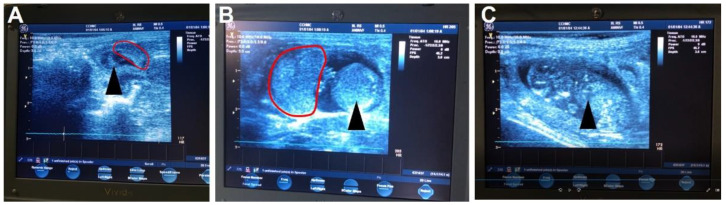
Ultrasound images of the guinea pig pregnancy. The earliest time to reliably detect a guinea pig pregnancy using an ultrasound is approximately gestational day 21, in which the most obvious signs of a fetus are the amniotic cavity and placenta (**A**). By mid-gestation (gestational day 30–35) the placenta and sub-placental region are visible along with the fetus (**B**) including a clear identification of the fetal heart (**C**). The black arrow identifies the developing fetus, pointing to the fetal heart in (**C**). The red outline indicates the placenta.

Whilst it is relatively easy to observe the estrus cycle of the guinea pig by monitoring the vaginal membrane, determining a successful pregnancy is more difficult. Guinea pigs carry weight around their abdomens making visual observations of pregnancy practically impossible until after gestational day 40. Furthermore, noticeable increases in maternal weight, that would suggest a pregnancy, do not occur until after mid-pregnancy and can vary depending on the litter size (Figure 3). Some have described methods of gently palpating the maternal abdomen as early as gestational day 15 to feel for conceptuses [21]. However, we have not been successful in using this methodology. Daily monitoring for vaginal membrane perforation after the female has been with the male and assumed pregnant is also not a viable technique. As our data shows, we have observed spontaneous perforation in the vaginal membrane after a successful mating in two out of the five pregnant animals (Figure 2, animal #3 and #5). This phenomenon has previously been described, with the original hypothesis that opening of the membrane during pregnancy was associated with fetal loss [22]. However, further studies concluded that perforation of the membrane during pregnancy was a natural process unique to the guinea pig and possibly driven by changes in estrogen and progesterone [17]. Our experience supports this conclusion as early pregnancy ultrasounds showed no evidence of fetal resorptions in the animals whose vaginal membranes perforated during pregnancy. In our experience, an ultrasound is the only definitive method for determining a pregnancy prior to gestational day 30 (Figure 4), but is limited to not being accurate before gestational day 21.

Integral to reproductive research is the ability to time mate animals in order to monitor and collect data at similar points throughout gestation. In this paper, we report an easy and reliable method of time mating guinea pigs through daily monitoring of the vaginal membrane. A significant advantage of the method is that this mating strategy can be performed by a single researcher and does not require overly specialized tools or equipment as well as negates the need to perform daily, time-consuming smears in order to track the estrus cycle. Coupled with an ultrasound as a secondary method to confirm the pregnancy, we have shown that through familiarizing ourselves with the changes that occur to the vaginal membrane, we can achieve a 100% successful pregnancy rate in a matter of weeks, which ultimately reduces the overall costs associated with animal husbandry and increases research success. 

## Data Availability

Data can be provided on request by the corresponding author.
